# The enhanced immunological activity of Paulownia tomentosa flower polysaccharide on Newcastle disease vaccine in chicken

**DOI:** 10.1042/BSR20190224

**Published:** 2019-05-03

**Authors:** Haifeng Yang, Ping Zhang, Xiaozhou Xu, Xiaolan Chen, Qingxin Liu, Chunmao Jiang

**Affiliations:** 1 Department of Animal Pharmacy, Jiangsu Agri-Animal Husbandry Vocational College, Taizhou, Jiangsu Province 225300, P.R. China; 2 Department of Animal Husbandry and Veterinary Medicine, Jinling Institute of Technology, Nanjing, Jiangsu Province 211169, P.R. China; 3 Department of Animal Science and Technology, Jiangsu Polytechnic College of Agricultural and Forestry, Zhenjiang, Jiangsu Province 212400, P.R. China

**Keywords:** cytokines, lymphocyte proliferation, Newcastle disease vaccine, Paulownia tomentosa flower polysaccharides, serum antibody titer

## Abstract

The extracts of *Paulownia tomentosa* (*P. tomentosa*) exhibit multiple pharmacological activities. In the present study, *P. tomentosa* flower polysaccharides (PTFP) were extracted by water decoction and ethanol precipitation, and the immunologic modulations of PTFP against Newcastle disease (ND) vaccine was investigated in chickens. The results showed that in a certain range of concentrations, PTFP treatment can dose-dependently enhance lymphocyte proliferation. Then, 280 14-days-old chickens were randomly divided into seven groups, and vaccinated with ND vaccine except blank control (BC) group. At the first vaccination, chickens were orally administrated with PTFP at concentration ranging from 0 to 50 mg/kg once a day for 3 successive days, and the BC group was treated with physiological saline. The lymphocyte proliferation rate, serum antibody titer, and levels of interferon-γ (IFN-γ) were respectively measured on 7, 14, 21, and 28 days after the first vaccination. The results showed that PTFP at the suitable doses could significantly promote lymphocyte proliferation, enhance serum antibody titer, and improve serum IFN-γ concentrations. Taken together, these data indicated that PTFP could improve the immune efficacy against ND vaccine in chickens, and could be as the candidate of a new-type immune adjuvant.

## Introduction

*Paulownia tomentosa* (*P. tomentosa*) is an extremely fast-growing tree species with multiple uses, and is widely cultivated in saline regions [1]. The bark, leaves, and flowers of *Paulownia* trees have been used in traditional medicine to treat infectious and inflammatory diseases in East Asia [2]. Pharmacological studies showed that the extracts from *P. tomentosa* could inhibit the protein tyrosine phosphatase 1B and α-glucosidase, which are important therapy targets of obesity and diabetes treatment [3], suppress the production of IL-6 and TNF-α in LPS-stimulated RAW 264.7 macrophages [2], and treat airway inflammation [4]. It has been also demonstrated that methanolic extracts of *P. tomentosa* flowers are active against EV71 of hand, foot, and mouth disease [5]. Multiple bioactive constituents from *P. tomentosa* species, including polyphenols, flavonoids, and polysaccharides, have been reported in previous studies, and polysaccharides are considered to be one of the major active ingredients in *P. tomentosa*. However, the effects of *P. tomentosa* flower polysaccharides (PTFP) on animal virus infectious diseases are rarely reported.

Immunologic adjuvant plays an important role in prevention of infectious disease with different types of vaccine, such as aluminum and oil adjuvant [1], but these chemical adjuvants commonly cause side effects, such as strong local stimulation and carcinogenesis [6,7]. Some chemical adjuvants failed to enhance the immunogenicity of weak antigen vaccine [8]. Therefore, it is urgent to study and develop a new-type immunologic adjuvant with high efficiency, low toxicity, and extensive resources. Chinese herbal medicines and their ingredients, as a new type of adjuvant, possess several advantages, such as reliable efficacy and low side effects [9]. Various herbal preparations have been shown to exert strong immuno-modulatory properties, and applied to chickens before or after vaccination; thus, the incidence of infectious diseases is decreased and animal immune response is increased [10].

In the present study, the PTFP were extracted from air-dried flowers of *P. tomentosa* by water decoction and ethanol precipitation. The immune-enhancing activities of PTFP were investigated by the experiments *in vitro* and *in vivo*. The purpose of the present study is to evaluate the adjuvant activity of PTFP, and screen out a candidate as a new immunologic adjuvant.

## Materials and methods

### Preparation of medicine

Dried-cultured *P. tomentosa* flowers militaris were obtained from the Bozhou Guoxin Pharmaceutical Co., Ltd (Anhui, China). The PTFP were prepared by water decoction and ethanol precipitation. Briefly, the dried cultured *P. tomentosa* flowers were extracted twice with the distilled water (2 h for the first time, 1 h for the second time), the decoction was filtered, merged, and condensed into 500 ml, then the concentrated liquid was centrifuged. The suspension was precipitated with 95% ethanol four times for a total of 12 h. After the supernatant was removed, the rest was centrifuged and concentrated to a specific volume under reduced pressure condition.The carbohydrate concentration (%) of total PTFP was 48 compared with D-glucose.

### Reagents

Improved RPMI-1640 (Thermo Fisher Scientific, U.S.A.) medium added with 100 IU/ml benzyl penicillin/streptomycin and 10% fetal bovine serum (FBS) was used for cell culture. Calcium and magnesium-free (CMF) phosphate-buffered saline (PBS, pH 7.4) was used to configure a 5 mg/ml 3-(4, 5-Dimethylthiazol-2-yl)-2, 5-diphenyltetrazolium bromide (MTT, GenView Co.) solution. Concanavalin-A (Con-A, Sigma, U.S.A.), a T-cell mitogen, configured to 100 mg/ml using RPMI-1640 medium. Dissolved Con-A and MTT solutions were filtered using a 0.22 µm membrane Millipore filter, and maintained at −20°C. DMSO reagent was commercially brought from Ling Feng Chemical Reagent Co., Ltd. (Shanghai, China). Peripheral blood lymphocyte separation medium for chicken (No. LTS1900C) was obtained from Hao Yang Biological Manufacture Co., Ltd (Tianjin, China).

### *In vitro* test

Safe concentrations of PTFP used for chicken peripheral lymphocytes were measured by MTT assay [11–13]. The result showed that the absorbance at 570 nm (A_570_) values of PTFP at 0.25–8 mg/ml were not significantly lower than those of corresponding control group. Therefore, its maximal safety concentration could be determined at 8 mg/ml.

PTFP were dissolved with RPMI-1640 into six gradient concentrations ranging from 0.25 to 8 mg/ml based on the above-mentioned safety concentration. Blood samples (5 ml) were collected from chickens and immediately transferred into aseptic capped tubes added with 3.8% sodium citrate solution, then diluted with 5 ml D-Hank’s, and the blood samples were mixed gently and layered on the surface of lymphocytes separation medium carefully. After centrifugation (20 min, 2000 rpm), a white cloud-like lymphocyte band was collected and washed with RPMI 1640 medium twice. The resulting pellet was re-suspended and diluted to 5 × 10^6^/ml with RPMI-1640 medium. The solutions were divided into two parts: one part was respectively incubated into 96-well plate without Con-A, 100 µl per well, and the other part was added with Con-A (at a final concentration of 10 µg/ml). At the same time, PTFP (0.25–8 mg/ml) was added (100 µl per well) with four replicates. In blank control (BC) group, cells were cultured with RPMI-1640 medium. In 0-dose PTFP group, cells were cultured with RPMI-1640 medium adding 20µl Con-A. The plates were incubated in a humid atmosphere with 5% CO_2_ at 37°C for 48 h. 44 h after incubation, 20 µl of MTT (5 mg/ml) was added into each well, followed by further incubation for 4 h. Then the plates were centrifuged at 1000×***g*** for 10 min at room temperature. The supernatant was removed carefully and 100 µl of DMSO was added into each well. The plates were shaken for 5 min to dissolve the crystals completely. The A_570_ was measured by microliter enzyme-linked immunosorbent assay reader [14,15].

### *In vivo* test

#### Experimental animals

One-day-old White Roman chickens, brought from Tangquan Poultry Farm (Nanjing, China) were housed in mesh cages (0.6 × 1 m) in a temperature-controlled 37°C room and lighted for 24 h at the initial period. The lighting time and environment temperature respectively were gradually adjusted to 12 h per day, and the room temperature was constantly maintained in the following days. Chickens were fed with commercial animal feed obtained from Jiangsu Academy of Agricultural Science (China). All animals were handled in compliance with the guidelines set forth by the Guide for the Care and Use of Laboratory Animals and according to the guidelines of the Institutional Animal Care and Use Committee.

#### Vaccine and antigen

Newcastle disease (ND) vaccine (Lasota strain, No. 170010301) and corresponding ND detection antigen used in present study were provided by Qingdao Yebio Bioengineering Co., Ltd (Shandong, China).

#### Experimental design

Two hundred and eighty 14-days-old chickens (average maternal antibody titer being 2.25log_2_) were randomly divided into seven groups. The chickens – except the BC group – were vaccinated with ND vaccine by nose and eye-dropping method, repeating vaccination at 28-days-old. At the same time as the first vaccination, the chickens in PTFP groups were orally administrated with PTFP at different doses (50, 25, 12.5, 6.25, and 3.125 mg/kg), while vaccination control (VC) and BC groups were given physiological saline, once a day for 3 successive days. On 7, 14, 21, and 28 days after the first vaccination, the changes of peripheral lymphocyte proliferation and serum antibody titers of the chickens were respectively determined by MTT assay and hemagglutination inhibition (HI) test [16,17], and the serum interferon-γ (IFN-γ) concentrations were determined by ELISA [15,18,19].

#### Peripheral lymphocyte proliferation assay

Peripheral lymphocytes assay was performed as previously described [6]. At indicated time point, blood samples (5 ml per chicken) were collected from heart, and then peripheral blood lymphocytes were isolated and performed as described above. The isolated lymphocytes were diluted to 2.5 × 10^6^/ml with RPMI-1640 complete medium, and 80 µl cell suspension was added into each well of 96-well plate supplemented with 20 µl Con-A. The following procedures were same as previous described. The light A_570_ was measured by microliter enzyme-linked immunosorbent assay reader as the index of lymphocytes proliferation *in vivo*.

#### Serum HI antibody assay

Blood samples (1.0 ml per chick) from brachial vein were allowed to clot respectively at 37°C for 2 h and 4°C for 1 h. Serum was separated by centrifugation for determination of HI antibody. The determination method was referred to the previous literature [16]. Micromethod was used to calculate HI, the mean titer expressed as reciprocal log2 value of the highest dilution [20]. To eliminate the influence of naturally raised antibody titer increased with age, the data were normalized with BC groups.

#### Serum cytokines concentration assay

Blood samples (2.0 ml per chick) from brachial vein were drawn into Eppendorf tube and allowed to clot at 37°C for 2 h. Serum was separated by centrifugation for determination of the cytokines concentrations. The concentrations of IFN-γ in serum were assayed by ELISA according to the kit’s instruction [18].

### Statistical analysis

Data in the present study are expressed as mean ± S.D. (standard deviation). Statistical differences of the lymphocytes proliferation, antibody titer, and serum cytokines concentrations among groups were analyzed by Duncan’s multiple range test. *P*<0.05 was regarded as significant statistical difference between groups.

## Results

### Effect of PTFP on lymphocytes proliferation rate *in vitro*

#### The alterations of lymphocyte proliferation rate with stimulation of PTFP

**Figure 1:**
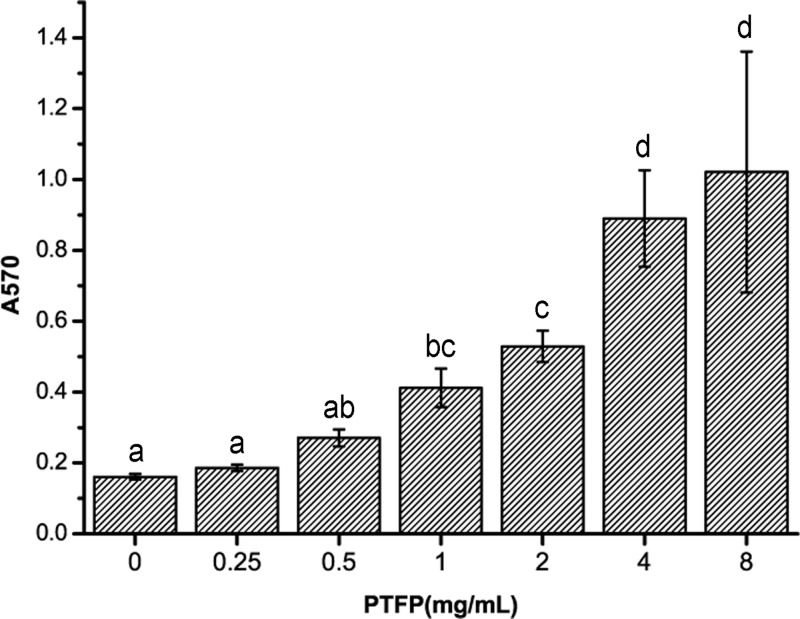
The effect of PTFP stimulation on the proliferation of chicken peripheral blood lymphocytes *in vitro* Column data marked without the same characters (**A–D**) differ significantly (*P*<0.05).

Effect of PTFP on the proliferation of chicken peripheral blood lymphocytes is shown in Figure 1. The relative proliferation rate (A_570_) of 1–8 mg/ml PTFP-treated lymphocytes are significantly increased compared with untreated cells (*P<*0.05). The relative proliferation rate of cells treated with PTFP at 0.25–0.5 mg/ml are also numerically higher than that of untreated cells (*P>*0.05). Therefore, 1–8 mg/ml PTFP treatment can promote chicken lymphocytes proliferation *in vitro*.

#### The alterations of lymphocyte proliferation rate with synergistic stimulation of PTFP and Con-A

**Figure 2:**
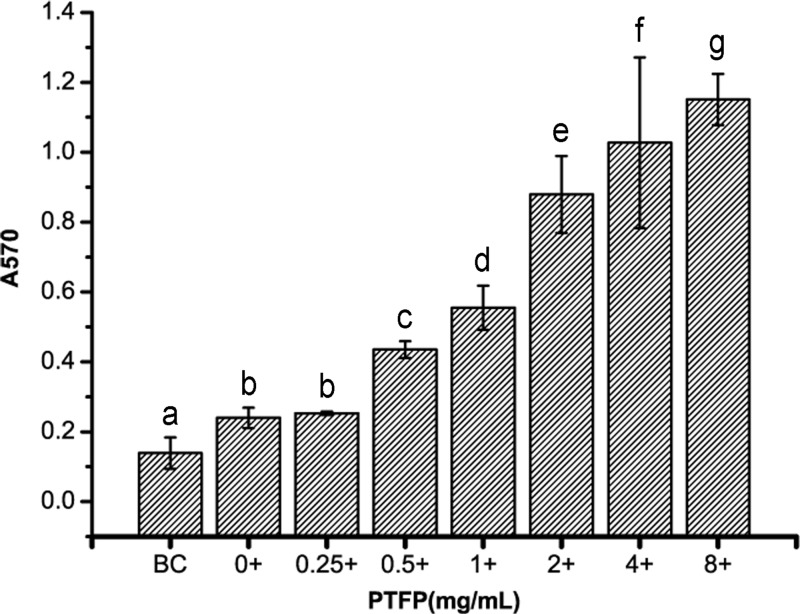
The effect of PTFP and Con-A synergistic stimulation on the proliferation of chicken peripheral blood lymphocytes *in vitro* BC was represented as cells untreated with Con-A or PTFP. ‘+’ means 10 mg/ml Con-A treatment. Column data marked without the same characters (**A–G**) differ significantly (*P*<0.05).

The effect of PTFP with Con-A on the *in vitro* proliferation of chicken peripheral blood lymphocytes is shown in Figure 2. Con-A treatment (10 mg/ml) could significantly accelerate the chicken lymphocytes proliferation compared with untreated cells. PTFP could synergistically enhance Con-A treated lymphocytes proliferation in a concentration-dependent manner. In addition, a lower dose (0.5 mg/ml) PTFP treatment can more effectively increase lymphocytes proliferation when applied with Con-A. Treatment of combination Con-A with PTFP markedly increase lymphocytes proliferation compared with PTFP alone, over a range from 1 to 8 mg/ml. These data indicate that PTFP – in combination with Con-A – exhibited the synergistic promotion effect of chicken peripheral lymphocyte proliferation.

### *Effect of PTFP on the lymphocyte proliferation in* chickens vaccinated with ND vaccine

**Figure 3:**
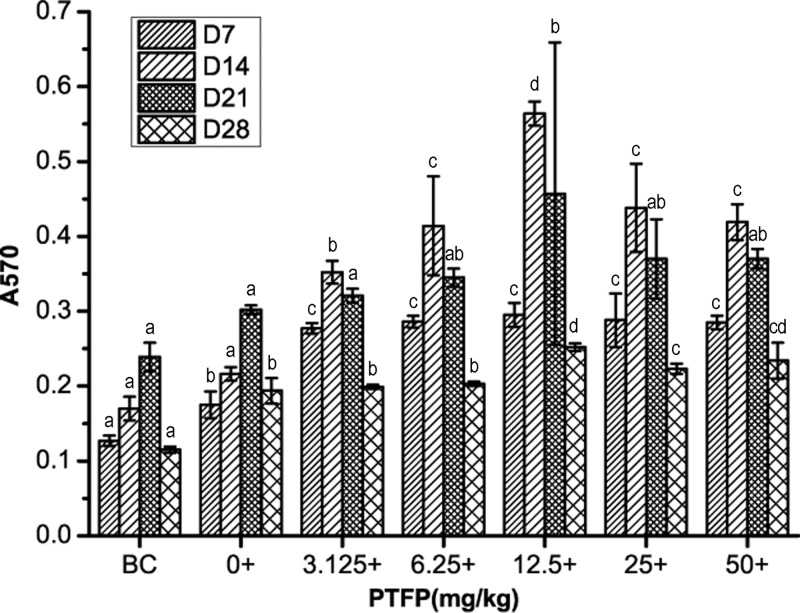
The effect of PTFP treatment on the proliferation of peripheral blood lymphocytes in chickens vaccinated with ND vaccine BC was represented as BC group using physiological saline instead of ND vaccine and PTFP. ‘+’ means 10 mg/ml Con-A treatment. Column data marked without the same characters (**A–D**) differ significantly at the same time point (*P*<0.05).

The relative lymphocytes proliferation of each group after exposure to ND vaccine alone or in combination with PTFP is shown in Figure 3. On day 7, after the first vaccination, vaccine alone enhanced peripheral lymphocyte proliferation compared with BC. The relative lymphocytes proliferation could be further increased when vaccinated in combination with FTFP at 3.125–50 mg/kg compared with the vaccine alone. Day 14–21 after the first vaccination, the A_570_ values are further increased at the corresponding concentrations of PTFP (3.125–50 mg/kg) compared with that on day 7, and then their vaccination levels gradually decrease with the increase in vaccination period. Notably, the relative lymphocytes proliferation rate of 12.5 mg/kg PTFP treatment at day 14 is the highest among total experimental groups (*P*<0.05). On Day 28, the A_570_ values of PTFP at 12.5–50 mg/kg are significantly higher than those of vaccine alone as well (*P*<0.05). Overall, 3.125–50 mg/kg PTFP treatment (best at 12.5 mg/kg) could further accelerate the promotion effects of relative lymphocytes proliferation triggered by ND vaccine.

### *Effect of PTFP on the serum antibody titer in* chickens vaccinated with ND vaccine

**Figure 4:**
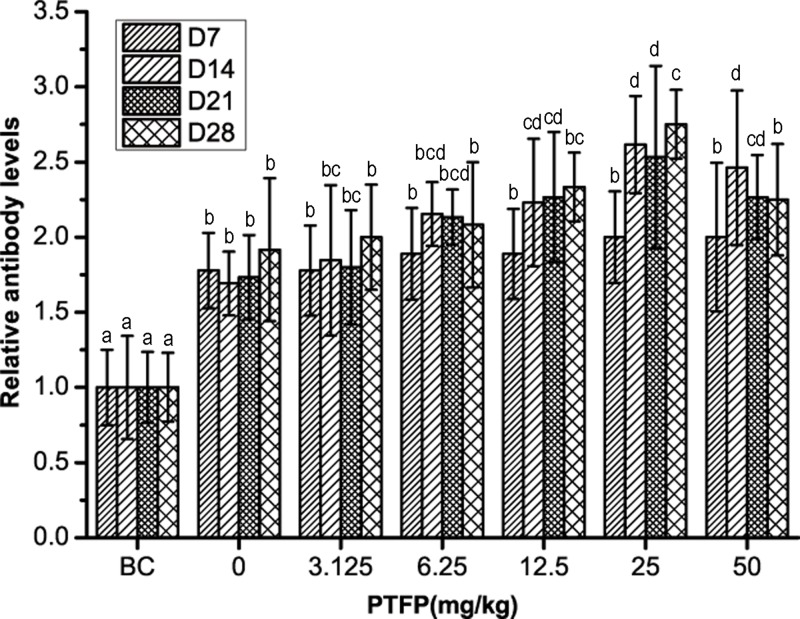
The effect of PTFP treatment on the serum antibody titers in chickens vaccinated with ND vaccine BC was represented as BC group treated with physiological saline instead of ND vaccine and PTFP. Column data marked without the same characters (**A–D**) differ significantly at the same time point (*P*<0.05). Group relative antibody levels (Day n) = Group antibody titers at Day n/BC group antibody titers at Day n.

The alteration of serum antibody titers after exposure to ND vaccine alone or in combination with PTFP is showed in Figure 4. Day 7 after the first vaccination, the serum antibody titers in all PTFP groups are not significantly higher than those in vaccine alone (*P>*0.05). On day 14 and 21, the antibody titers are markedly enhanced when exposed to PTFP at 12.5–50 mg/kg compared with vaccine alone (*P<*0.05). With the increase in vaccination period, the antibody titers decrease in all groups. On day 28 the PTFP at only 25 mg/ml show the significant change of antibody titers compared with vaccine alone (*P<*0.05). In addition, the antibody titers in 25 mg/kg PTFP treatment group on day 28 keep the highest level among all groups. Generally, PTFP could heighten and maintain the serum antibody titers in ND vaccination chicken.

### *Effect of PTFP on the serum levels of IFN-γ in* chickens vaccinated with ND vaccine

**Figure 5:**
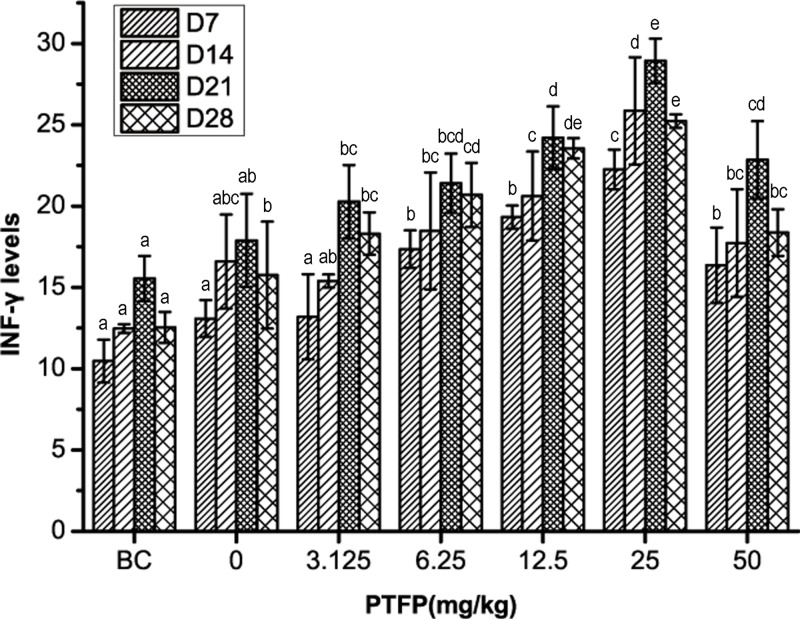
The effect of PTFP treatment on serum levels of IFN-γ in chickens vaccinated with ND vaccine BC was represented as BC group treated with physiological saline instead of ND vaccine and PTFP. Column data marked without the same characters (**A–E**) differ significantly at the same time point (*P*<0.05).

The serum levels of IFN-γ in each group is shown in Figure 5. In ND vaccination chicken, serum levels of IFN-γ are much higher than that in BC group. The treatment of PTFP at 6.25–50 mg/kg could significantly enhance the IFN-γ concentrations compared with the vaccine alone (*P<*0.05). On day 14, the IFN-γ concentrations in 25 mg/kg PTFP treatment group is significantly higher than that in the vaccine alone (*P<*0.05). On day 21, the IFN-γ levels in 12.5–50 mg/kg PTFP treatment groups are significantly higher than that of vaccine alone (*P<*0.05). Taken together, 12.5–50 mg/kg PTFP treatment (best at 25 mg/kg) could significantly increase the serum levels of IFN-γ in chickens vaccinated with ND vaccine.

## Discussion

Lymphocytes are the important components of the immune system. Lymphocyte proliferation rate is a direct indicator of the state of cellular immunity [21,22], and is a good criteria to study the immunomodulatory activity of drugs [23]. MTT assay is a classical method to determine the cell proliferation rate, and there exists a positive correlation between the A_570_ value and cell number [21]. Therefore, in the current study, MTT assay was used to analyse the lymphocyte cell viability after exposure to PTFP.

As novel immune adjuvants against infections, extracted polysaccharides derived from Chinese medicinal herbs attract researchers’ attention by several advantages [24,25]. Yang et al. [26] have reported that extracts from *Astragalus membranaceus* and *Scutellaria baicalensis* can increase type 1 helper T (Th1)-type cellular immune response in UV-attenuated *Toxoplasma gondii*-infected mice model. Wang et al. [27] have showed that the polysaccharide from *Paulownia fortunei* flowers can promote cellular and humoral immunity in chickens vaccinated with ND vaccine through increasing the levels of IL-2 and IFN-γ, duodenal sIgA content as well. Additionally, Zhao et al. [28] have reported that polysaccharides from the rhizome of *Atractylodis macrocephalae Koidz* (RAMPS) could be an immunopotentiator in chickens vaccinated with ND vaccine, which enhances serum HI antibody titer and improves the percentages of CD4^+^ and CD8^+^ T cells. In our previous study, we also reported that *Ganoderma lucidum* polysaccharide (GLP) could be an immunomodulator in chickens vaccinated with ND vaccine through promoting lymphocyte proliferation and elevating serum antibody titer [6]. The purpose of the present study is to further investigate the immune protection role of PTFP in chickens against ND vaccine.

In the present study, *in vitro* test showed that 1–8 mg/ml PTFP stimulation could promote the chicken peripheral blood lymphocyte proliferation in a concentration-dependent manner. Besides, 0.5–8 mg/ml PTFP stimulation also enhanced relative lymphocyte proliferation rate when applied with Con-A, implying that PTFP synergized with Con-A in promoting the proliferation of lymphocytes. Additionally, in the *in vivo* studies, the results of the immune response test showed that 12.5 mg/kg PTFP treatment in chickens vaccinated with ND vaccine can most effectively accelerate lymphocyte proliferation rate. This suggested that PTFP could enhance the cellular immune responses against ND vaccine and improve the adjuvant activity. Accordingly, the experimental results manifested that the PTFP at suitable concentrations could enhance the function of immune competent cells.

Humoral immunity mediated by antibodies are mainly triggered by B lymphocytes [29], and the antibody levels are good markers to reflected humoral immune function in bird species [30]. So the antibody titer is the indicator for humoral immunity and plays a critical role in the host’s defense against infectious disease [31]. In the present study, the antibody titers in PTFP-treated groups were significantly higher than that in BC or vaccine group, especially exposed to the PTFP at 25 mg/kg. These indicated that PTFP not only could promote lymphocyte proliferation, but also enhance the humoral immunity response against ND vaccine.

IFN-γ is secreted by Th1 cells, which are involved in cellular immunity [32]. As considerable immune correlation factors of Th1 cells, IFN-γ can indicate the basic status of cellular immunity of organism to certain extent [13]. Therefore, the alteration of serum IFN-γ levels is one of the important reflector indicating immune response against ND vaccine in chickens. In order to further investigate the adjuvant activity of PTFP, the effect of PTFP on secretion of IFN-γ was studied. The results revealed that the serum IFN-γ levels in PTFP treated-groups are significantly higher than that in the vaccine alone or BC group at most time points, especially exposure to PTFP at 12.5–25 mg/kg. Similarly, Yang et al. [33] have showed that polysaccharides extracted from tetraploid *Echinacea purpurea* at 0.0312 mg/ml possess the highest acceleration of lymphocyte proliferation and secretion of IFN-γ in mice lymphocyte, the same with polysaccharides extracted from diploid *Echinacea purpurea* at 0.125 mg/ml. In our previous study, we also found that GLP at 115 µg/ml dose group have a stronger promotion effect on IFN-γ mRNA expression in chickens than at 112.5 and 450 µg/ml dose groups [6]. Our results indicated that PTFP at the suitable doses could promote the IFN-γ secretion, thus enhance immunity. However, further research is required to address whether PTFP could alter concentrations of other cytokines (e.g., IL-2, IL-10, and IL-13).

In conclusion, PTFP not only enhanced lymphocyte proliferation and serum antibody titers in chickens inoculated with ND vaccine, but also promoted the levels of IFN-γ in serum. Additionally, 12.5–25 mg/kg dose usage of PTFP in chickens vaccinated with ND vaccine may have the best efficiency to enhance immune response. Therefore, PTFP exhibited immunological adjuvant potential in regulating cellular and humoral immunities.

## Abbreviations

A570: absorbance at 570 nm
BC: blank control
Con-A: concanavalin A
GLP: *Ganoderma lucidum* polysaccharide
HI: hemagglutination inhibition
IFN-γ: interferon-γ
IL: interleukin
LPS: lipopolysaccharide
MTT: 3-(4, 5-dimethylthiazol-2-yl)-2, 5-diphenyltetrazolium bromide
ND: Newcastle disease
PTFP: *P. tomentosa* flower polysaccharides
*P. tomentosa*: *Paulownia tomentosa*
sIgA: secretory immunoglobulin A
Th1: type 1 helper T
TNF-α: tumor necrosis factor α
